# Substrate recognition determinants of human eIF2α phosphatases

**DOI:** 10.1098/rsob.210205

**Published:** 2021-12-01

**Authors:** George Hodgson, Antonina Andreeva, Anne Bertolotti

**Affiliations:** MRC Laboratory of Molecular Biology, Francis Crick Avenue, Cambridge CB2 0QH, UK

**Keywords:** phosphatase, PP1, PPP1R15, integrated stress response, eIF2α phosphorylation

## Abstract

Phosphorylation of the translation initiation factor eIF2α is a rapid and vital cellular defence against many forms of stress. In mammals, the levels of eIF2α phosphorylation are set through the antagonistic action of four protein kinases and two heterodimeric protein phosphatases. The phosphatases are composed of the catalytic subunit PP1 and one of two related non-catalytic subunits, PPP1R15A or PPP1R15B (R15A or R15B). Here, we generated a series of R15 truncation mutants and tested their properties in mammalian cells. We show that substrate recruitment is encoded by an evolutionary conserved region in R15s, R15A^325–554^ and R15B^340–639^. G-actin, which has been proposed to confer selectivity to R15 phosphatases, does not bind these regions, indicating that it is not required for substrate binding. Fragments containing the substrate-binding regions but lacking the PP1-binding motif trapped the phospho-substrate and caused accumulation of phosphorylated eIF2α in unstressed cells. Activity assays in cells showed that R15A^325–674^ and R15B^340–713^, encompassing the substrate-binding region and the PP1-binding region, exhibit wild-type activity. This work identifies the substrate-binding region in R15s, that functions as a phospho-substrate trapping mutant, thereby defining a key region of R15s for follow up studies.

## Graphical abstract

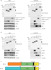

## 1. Introduction

Phosphorylation of the alpha subunit of the heterotrimeric translation initiation factor 2 (eIF2α) on serine 51 [1] is an evolutionarily conserved defence mechanism essential for cell survival and organismal fitness [2,3]. It results in a transient reduction of bulk translation activity concomitant with a selective increase in translation of some transcripts [1,4]. In mammals, the phosphorylation levels of eIF2α are tuned to cellular needs through the antagonistic actions of four different eIF2α kinases, PKR, HRI, PERK and GCN2 and two eIF2α phosphatases [1]. The latter are composed of the catalytic subunit PP1 bound to one of two related non-catalytic subunits PPP1R15A [5,6] or PPP1R15B [7]. Because PP1 controls a large number of protein dephosphorylation events, it was initially proposed to be non-selective [8,9]. However, while PP1 dephosphorylates many substrates *in vitro* [8,10], it is not free in cells, but bound to one or more proteins [9,11]. Non-catalytic subunits of PP1 phosphatases are dissimilar but they bind PP1 using conserved short linear motifs, the most common of which is RVxF, which docks to a surface of PP1 opposite the catalytic site [12].

Previous studies have revealed the importance of various regions of R15A using cellular readouts. The function of human R15A was elucidated on the basis of its sequence similarity to the protein ICP34.5 from herpes simplex virus, which blocks the host response to infection by recruiting PP1 to dephosphorylate eIF2α [13]. Further analyses led to the discovery that PP1 binding is through the carboxy-terminal region of ICP34.5, which harbours an RVxF motif [14]. The carboxy-terminal region of ICP34.5 is essential for its function and can be replaced by the homologous region of R15A [14]. A genetic screen in mammals later identified a fragment of R15A on the basis of its ability to repress expression of *Chop*, a target of the integrated stress response (ISR) [5]. This fragment, called A1, consists of hamster R15A^292–590^ (homologous to human R15A^310–658^) [5]. Structure–function analyses revealed that the first 200 amino acids of R15A are not required for the ISR-repressing activity of the overexpressed protein but important for its localization to the ER [5,15,16]. By contrast, the carboxy-terminal region is essential for function [5,15]. Expression of the carboxy-terminal 70 amino acids was deemed to be active in repressing ISR target genes, although the activity of this small fragment was markedly decreased relative to hamster R15A^292–590^ or full-length mouse protein [5]. Similar observations were made following overexpression of human R15 fragments in yeast expressing human eIF2α and PP1: full-length and R15A^420–674^ were equally active in rescuing from the toxicity resulting from overexpression of an eIF2α kinase [17]. In the same system, R15A^513–674^ had residual activity [17]. Importantly, active A1 is homologous to full-length ICP34.5 [13]. Thus, diverse findings have revealed that the first 200 amino acids of R15A are dispensable for the function of the overexpressed protein.

We previously reconstituted human eIF2α phosphatases with recombinant PP1 and large fragments of recombinant R15A and R15B homologous to ICP34.5 [18]. These complexes dephosphorylate eIF2α, but not other substrates, establishing that they recapitulated the function and selectivity of the native holoenzymes [18]. In this minimal system, composed of R15, PP1 and the amino-terminal fragment of eIF2α, R15s recruit the substrate, providing substrate selectivity to the holoenzyme [18]. The carboxy-terminal part of R15, with the RVxF motif, recruits PP1 but the resulting complex is not selective for eIF2α [18], in agreement with previous studies [15,19,20]. The eIF2α binding region mapped to the middle of R15s and is essential for selectivity of the reconstituted enzymes [18]. Another study also reported binding of eIF2α to the middle region of a recombinant R15A [19]. According to these findings, a selective holophosphatase is a split enzyme that requires the assembly of two components, one harbouring substrate binding and the other providing the catalytic function, both being essential [10].

In an alternative model, G-actin was proposed to provide substrate specificity to the otherwise unselective recombinant eIF2α phosphatases reconstituted with PP1 and the carboxy-terminal 70 amino acid region of R15s [20]. This model stems from the observation that overexpression of this region is sufficient to decrease expression of *CHOP*, an ISR target [5]. However, its activity is marginal compared to longer fragments [5]. Moreover, *in vitro*, this fragment does not confer substrate selectivity to the enzyme, which has similar properties to PP1 alone and dephosphorylates various substrates [20,21]. This prompted the search for a selectivity factor and left two open questions: what is the function of R15s? And why do humans have 674 and 713 amino-acid-long R15A and R15B proteins? Actin co-precipitates with overexpressed GFP-tagged R15A [22]. A crystal structure of a recombinant complex comprising of PP1, a carboxy-terminal 30 amino acid region of R15B, and G-actin was reported [20]. Actin was proposed to confer substrate selectivity in this *in vitro* system [20]. The biological relevance of actin binding for substrate recruitment has not been examined so far.

The two current models for eIF2α phosphatases selectivity agree that another factor is required in addition to the 70 amino acid PP1-recruiting region. One model proposes that selective substrate recruitment requires longer fragments of R15s [18], and the other is dependent on G-actin [20]. It is important to establish the requirement of G-actin in substrate recruitment by eIF2α phosphatases not only because these enzymes are central controllers of cellular fitness and drug targets [18,23–25], but also because such an advance could provide principles for elucidating substrate recognition of the many uncharacterized PP1 holoenzymes. The two current models for selectivity of the eIF2α phosphatases have been generated using recombinant proteins. Because R15s are predicted to be intrinsically disordered, recombinant proteins may not necessarily have biologically relevant folds and properties. To circumvent this issue, we investigated the substrate-binding requirements of R15s in cell-based assays and identified that their middle regions are responsible for substrate recruitment in absence of G-actin.

## 2. Results

**Figure 1.**
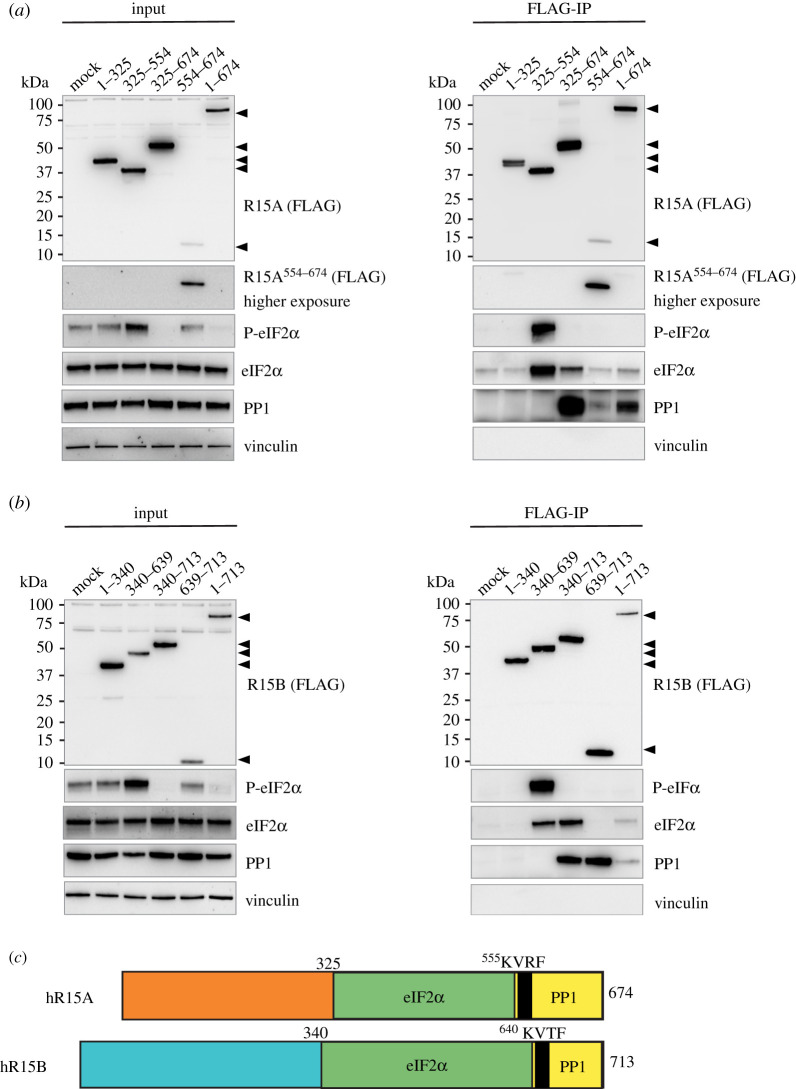
Phospho-substrate-trapping by the middle region of R15s. (*a*,*b*) R15 constructs were transfected into HEK 293T cells (input) and immunoprecipitated using anti-FLAG M2 magnetic beads (FLAG-IP). Samples were eluted from the beads by boiling in LDS and eluates were separated on a 4–12% Bis–Tris Plus gel. Proteins were detected by immunoblotting with FLAG, P-eIF2α, total eIF2α, PP1 and vinculin antibodies. Representative results of at least three experiments are shown. (*c*) A cartoon summary of the modular binding of eIF2α and PP1 to both R15A and R15B.

To identify functional regions in R15s, we generated a series of truncation mutants that were FLAG-tagged on their amino-termini. They were based on homology with the shorter viral protein ICP34.5, as well as clone A1 (electronic supplementary material, figure S1). Overexpressed R15A derivatives were immunoprecipitated with anti-FLAG antibodies (figure 1*a*). PP1 was found to interact with the R15A derivatives that encompassed residues 554–674, which contains the RVxF motif (figure 1*a*; electronic supplementary material, figure S2). Note that the antibody used to reveal PP1 in these experiments detects all three PP1 paralogues. eIF2α was enriched in the R15A^325–554^ and R15A^325–674^ pulldowns as well as in the pulldown of the full-length R15A^1–674^, albeit to a lesser extent (figure 1*a*; electronic supplementary material, figure S2). We found no enrichment of eIF2α upon immunoprecipitation of R15A^1–325^ or R15A^554–674^ (figure 1*a*; electronic supplementary material, figure S2). This reveals that R15A^325–554^ encodes the substrate recruitment region.

We next generated homologous truncation mutants of R15B (figure 1*b*). R15B derivatives containing its carboxy-terminal region captured PP1 when immunoprecipitated (figure 1*b*; electronic supplementary material, figure S3). By contrast, eIF2α was captured by R15B fragments (R15B^340–639^ and R15B^340–713^) that contained its middle region (figure 1*b*; electronic supplementary material, figure S3). R15B^639–713^ co-precipitated PP1 but not eIF2α (figure 1*b*; electronic supplementary material, figure S3). Conversely, R15B^340–639^ was bound to large amounts of eIF2α in absence of PP1 (figure 1*b*; electronic supplementary material, figure S3). Full-length R15B captured both eIF2α and PP1, although this was weaker than for the respective fragments (figure 1*b*; electronic supplementary material, figure S3). These experiments reveal that the binding of eIF2 and PP1 on R15B is not overlapping: R15B^340–639^ binds eIF2α while the carboxy-terminal region R15B^639–713^ binds PP1 (figure 1*b*; electronic supplementary material, figure S3). Thus, R15s are modular proteins with their carboxy-termini binding PP1, while the substrate recruitment region is encoded by a distinct region in the middle of the proteins (figure 1*c*).

The finding that in cells, the middle regions of R15s stably bind the substrate without binding the catalytic subunit raised the possibility that they might function as phospho-substrate trapping mutants. How PP1 holoenzymes selectively recognize their substrate is largely unknown, but structural information of MYPT-PP1 [26] and Phactr1/PP1 [27] has revealed that substrate binding is achieved by a composite surface between PP1 and the non-catalytic subunits. Thus, it has been challenging to design substrate trapping mutants for PP1 holoenzymes, because of their oligomeric nature. One approach to circumvent this problem has been to express covalent fusions of non-catalytic subunits with a catalytically compromised PP1 [28]. To examine if truncated R15s could trap the phosphorylated substrate, we monitored P-eIF2α in R15s immunoprecipitates. P-eIF2α was dramatically enriched in pulldowns of the middle region of R15s, R15A^325–554^ and R15B^340–639^, but no other fragments (figure 1*a*,*b*; electronic supplementary material, figures S2 and S3). This demonstrates that these middle fragments encode the substrate-recruiting region and because they lack the ability to recruit the catalytic subunit, they trap it in its phosphorylated form. Interestingly, expression of the various truncated fragments of R15s altered the levels of P-eIF2α detected in whole-cell lysates. This will be discussed in a later section.

**Figure 2.**
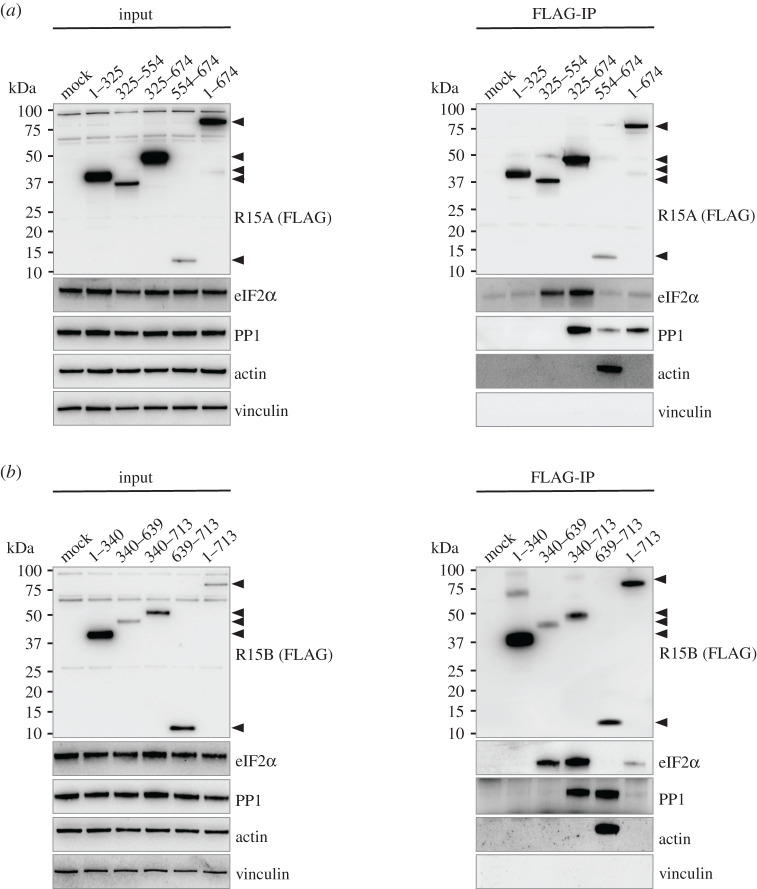
Actin binding is not required for substrate recruitment by the middle region of (*a*) R15A or (*b*) R15B. R15 constructs were transfected into HEK 293T cells (input) and immunoprecipitated using anti-FLAG M2 magnetic beads (FLAG-IP). Samples were eluted by boiling in LDS and eluates were separated on a 4–12% Bis–Tris Plus gel. Proteins were detected by immunoblotting with FLAG, total eIF2α, PP1, actin and vinculin antibodies. Representative results of at least three experiments are shown.

Next we examined if substrate recruitment by eIF2α phosphatases depends on actin in cells. We found that actin was significantly enriched following immunoprecipitation of R15A^554–674^ (figure 2*a*; electronic supplementary material, figure S2), in agreement with a previous report [22]. The capture of actin by this fragment was efficient despite the fact it expressed less than others (figure 2*a*; electronic supplementary material, figure S2). eIF2α was not detected in R15A^554–674^ pull downs (figure 2*a*; electronic supplementary material, figure S2). Conversely, the fragments that recruited the substrate did not pull-down actin (figure 2*a*; electronic supplementary material, figure S2). The fragments binding eIF2α were expressed at higher levels than the carboxy-terminal fragment co-precipitating actin (figure 2*a*). Thus, the absence of actin in the immunoprecipitates of R15A^325–554^ and R15A^325–674^ was not caused by insufficient capture of the baits (figure 2*a*; electronic supplementary material, figure S2). This reveals that the substrate binds without actin. Full-length R15A did not capture actin (figure 2*a*; electronic supplementary material, figure S2). Similar observations were made with R15B fragments. Actin was significantly captured following immunoprecipitation of R15B^639–713^, which was expressed and captured at levels comparable to other fragments (figure 2*b*; electronic supplementary material, figure S3). The middle region of R15B (R15B^340–639^) captured eIF2α, in absence of actin (figure 2*b*; electronic supplementary material, figure S3). These findings establish that that actin is not required for substrate recruitment.

**Figure 3.**
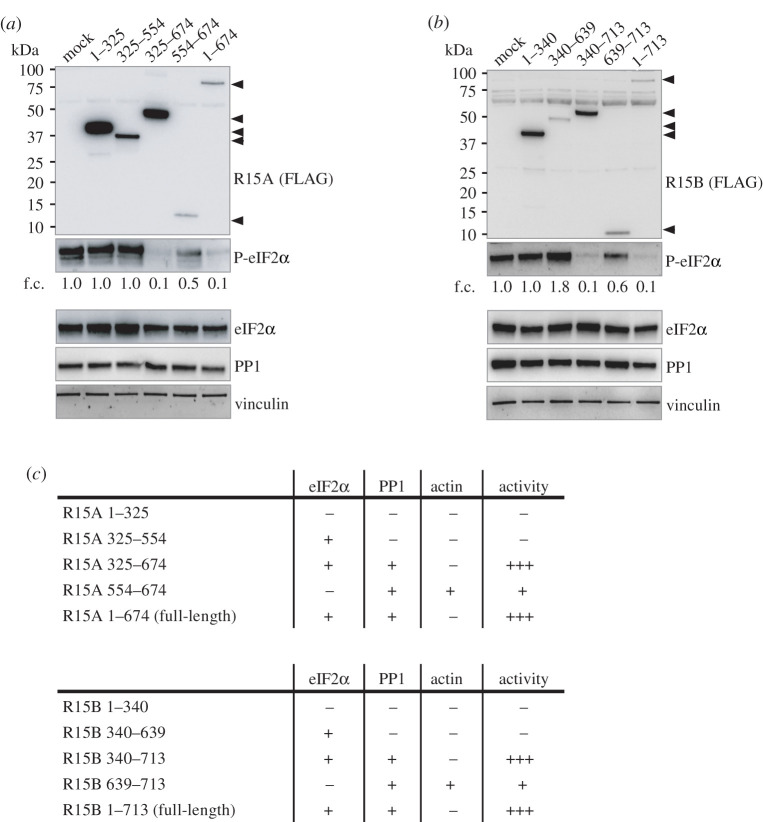
Activity of the various R15 constructs dephosphorylating P-eIF2α. (*a*,*b*) R15 constructs were transfected into HEK 293T cells, such that cells were overconfluent before lysis, providing high basal eIF2α phosphorylation to asses R15 construct activity. Proteins were detected by immunoblotting with FLAG, P-eIF2α, total eIF2α, PP1 and vinculin antibodies. Representative results of at least three experiments are shown. f.c.: fold changes relative to mock transfection. (*c*) Table summarizing the properties of R15 truncations.

We next tested the ability of R15 fragments to dephosphorylate eIF2α in cells exposed to nutrient stress to induce high levels of eIF2α phosphorylation [1]. Overexpression of full-length R15A^1–674^ led to a robust decrease in the levels of phosphorylated eIF2α (figure 3*a*). Overexpression of R15A^325–674^ also led to nearly complete dephosphorylation of eIF2α (figure 3*a*). Similarly, full-length R15B^1–713^ as well as R15B^340–713^ were highly active (figure 3*b*). The amino-terminal regions of R15s, which do not bind the substrate nor PP1, as well as R15A^325–554^ and R15B^340–639^, which bind the substrate but not PP1, did not decrease phosphorylation of eIF2α (figure 3*a*,*b*). These results are in agreement with the properties of the recombinant holoenzymes [18]. Intriguingly, overexpression of the carboxy-terminal fragment of R15A or R15B slightly but reproducibly decreased eIF2α phosphorylation (figure 3*a–c*; electronic supplementary material, figure S4*b*).

To enable direct comparison with previously published fragments, we generated R15A^533–624^ used in [21], the hamster A1 fragment [5], as well as R15A^325–636^ [18] (electronic supplementary material, figure S4*a*), and compared their properties with the variants generated in this study. Overexpressed A1 and R15A^325–636^ decreased eIF2α phosphorylation to background levels (electronic supplementary material, figure S4*b*), similar to R15A^325–674^ and R15B^340–713^, as well as full-length R15s. By contrast, R15A^533–624^ had a marginal effect, similar to R15A^554–674^ (electronic supplementary material, figure S4*b*). A1, R15A^325–636^ and R15A^325–674^ bound eIF2α as well as PP1, unlike R15A^533–624^ and R15A^554–674^, which bound PP1 but not eIF2α (electronic supplementary material, figure S4*c*). These results reveal that full activity of all fragments tested correlates with substrate and PP1-binding capacity. This work defines R15A^325–674^ and R15B^340–713^ as active fragments, which bind both eIF2α and PP1 (figure 3*c*).

The activity of the R15s derivatives was also evident in unstressed cells, which had lower, yet detectable levels of P-eIF2α (figure 1*a*,*b*). Expression of full-length R15s, as well as R15A^325–674^ and R15B^340–713^ led to complete dephosphorylation of eIF2α (figure 1*a*,*b*). No other fragment exhibited such activity. In this experimental context, expression of the carboxy-terminal regions of R15s had no notable activity (figure 1*a*,*b*). Importantly, expression of the R15A^325–554^ and R15B^340–639^ fragments caused an increase in eIF2α phosphorylation in unstressed cells (figure 1*a*,*b*). This is because these fragments trap the phosphorylated substrate (figure 1*a*,*b*). Such an activity was not revealed in stressed cells, with high (perhaps saturated) levels of eIF2α phosphorylation (figure 3*a*,*b*). This work identifies R15A^325–554^ and R15B^340–639^ as phospho-substrate trapping mutants.

## 3. Discussion and opening up

We generated a series of truncation variants of R15A and R15B to study their properties in mammalian cells. We find that the amino-terminal 300 amino acids are not required for recruitment of eIF2α, PP1 or eIF2α dephosphorylation. We show that R15A^325–554^ and R15B^340–639^ are necessary and sufficient for the recruitment of eIF2α. Substrate recruitment by these regions does not require actin.

While studies in cells provide a coherent body of information on the function of various regions of R15s, studies conducted *in vitro* with recombinant proteins have generated two models. In one model, substrate selectivity is provided by G-actin [20]. In the second model, substrate selectivity is provided by the middle region of R15 [18]. Here, we compared the properties of these protein fragments in cells. As shown before [5,6,15,19], we observed here that the carboxy-terminal region of the proteins is necessary and sufficient to bind PP1. We show that substrate binding required the middle region of R15s. This finding is in agreement with an earlier study of R15A in cells [19] and an *in vitro* study [18]. This region is evolutionarily conserved and present in ICP34.5 and clone A1.

We also assessed the requirement of actin for substrate recruitment in cells. While we validate the observation that the carboxy-terminal region of R15 is a strong actin binder [20,22], this property is restricted to short carboxy-terminal fragments of R15s and may be neomorphic. Supporting this possibility, the small carboxy-terminal fragments of R15s diffuse into the nucleus [15], unlike the full-length proteins, which localize to the endoplasmic reticulum. Regardless, we find that the middle region of R15, which lacks the canonical actin-binding motif described in the carboxy-terminal regions of R15s [20], binds eIF2 in absence of actin. This work defines a functionally important region for follow up studies.

Testing the activity of the various R15 truncation derivatives toward dephosphorylation of eIF2α yielded a coherent dataset. For high activity, R15 fragments need to contain the middle region of the protein binding the substrate and the carboxy-terminal PP1-binding region (figure 3*c*). These active fragments are homologous to clone A1 identified in an ISR suppressor screen [5]. The amino-terminal region is not required for activity in cells (figure 3*c*) and *in vitro* [18], but important for subcellular localization [5,15,16]. When expressed alone, the carboxy-terminal 70 amino acids of R15 bind PP1 and have residual activity, only detectable in stressed cells with high levels of phosphorylated eIF2α. The reason for this remains unclear. The activity of the various derivatives studied here explains the properties of similar recombinant fragments in a minimal *in vitro* system composed of R15, PP1 and the amino-terminal region of the substrate [18]: PP1 can dephosphorylate eIF2α but its activity is enhanced by the middle region of R15 which serves as a selective substrate receptor.

R15A and R15B are evolutionarily conserved and long proteins of 674 and 713 amino acids, respectively. While their carboxy-terminal 70 amino acid domain is sufficient to bind PP1 and actin, it is anticipated that other regions of the protein ought to be required for its function. No structural studies have been conducted so far with regions other than the generic PP1-binding carboxy-terminal regions of R15. Complexes composed of PP1 and a carboxy-terminal 30 amino acids of R15s are referred to as holoenzymes [15,19,20]. The crystal structure of R15B^630–701^ bound to PP1 has revealed that R15B binds PP1 via its RVxF motif in the canonical way [20]. A follow-up crystallographic and cryoEM study has been recently published where an amino-terminal fragment of eIF2α was added, as well Dnase1 to stabilize G-actin [29]. G-actin makes contact with R15A and this occurs via its promiscuous pocket that is known to bind a vast range of diverse proteins, suggesting competition between actin interactors [30]. These structural models show no direct contact between G-actin and eIF2α amino-terminal region or PP1 [29]. Whether impairment of actin binding reduces substrate recruitment has not been assessed. Therefore, how actin impacts on eIF2α signalling remains to be investigated.

The full-length R15s capture less substrate than shorter fragments. This is expected because enzymes do not form stable complexes with their substrates. However, it is not clear why the full-length R15s capture less PP1 than truncated fragments containing the RVxF motif. While the stoichiometry of interactions cannot be assessed by co-immunoprecipitations followed by immunoblots, the amount of PP1 in the immunoprecipitates of full-length R15B is low suggesting that in these experiments, only a fraction of overexpressed R15B engages with PP1. R15A full-length appears more efficient at capturing PP1, probably because of its higher affinity for PP1. Indeed, experiments with recombinant proteins have revealed that the affinity of PP1 is approximately 10-fold higher for R15A than R15B [18]. Because many non-catalytic subunits in cells compete for the same pool of PP1, assembly of PP1 holoenzymes is likely to be regulated. Recently, it was found that some cullin-ring ligases, which are conceptually similar to PP1 phosphatases in sharing catalytic subunits, require substrate engagement for assembly [31]. Thus, it is conceivable that the binding of PP1 to R15s is not constitutive but driven by the substrates or other modes of regulation. This is an interesting topic for future investigations.

The catalytic mechanism of PP1 dephosphorylation was elucidated more than two decades ago [32,33] yet there are only a couple of examples showing how non-catalytic subunits of phosphatases can contribute to substrate recruitment [26,27]. Here, we identify a domain of R15s that can stably bind the substrate in absence of the catalytic subunit and functions as a phospho-substrate-trapping mutant. Such mutants lacking the PP1-binding region can be designed with any non-catalytic subunits and used to identify substrates. Importantly, this work provides the basis for future detailed studies on substrate recruitment by R15s.

## 4. Material and methods

### 4.1. Cloning of R15 variants

All R15 derivatives were cloned from R15A/B human cDNA into the mammalian expression vector pXJ41 [34], which contains a single amino-terminal FLAG tag, using the Gibson Assembly method [35]. Clone A1 was cloned from haA1.pBABEpu. Primer sequences used are available in electronic supplementary material, table S1.

All R15 constructs cloned into the PXJ41 mammalian expression vector, harbouring a single amino-terminal FLAG tag.

**Table:** 

R15A constructs	species	boundaries based upon:
1–325	human	conservation and Carrara *et al*. [18]
325–554	human	conservation and Carrara *et al*. [18]
325–636	human	Carrara *et al*. [18]
325–674	human	conservation and Carrara *et al*. [18]
554–674	human	conservation and Carrara *et al*. [18]
1–674 (full-length)	human	conservation and Carrara *et al*. [18]
533–624	human	Chen *et al*. [20] and Crespillo-Casado *et al*. [21]
A1292–590	hamster	Novoa *et al*. [5] corresponding to 310–658 human R15A

**Table:** 

R15B constructs	species	boundaries based upon:
1–340	human	conservation and Carrara *et al*. [18]
340–639	human	conservation and Carrara *et al*. [18]
340–713	human	conservation and Carrara *et al*. [18]
639–713	human	conservation and Carrara *et al*. [18]
1–713 (full-length)	human	conservation and Carrara *et al*. [18]

### 4.2. Cell culture

Human embryonic kidney 293T cells (HEK 293T) were grown in a humidified incubator with 5% CO_2_ at 37°C. Cells were maintained in Dulbecco's modified Eagle's media (Sigma, D5796) supplemented with 100 U ml^−1^ penicillin and 100 µg ml^−1^ streptomycin (Gibco, 15140122), 2 mM l-glutamine (Gibco, 25030) and 10% fetal bovine serum (Gibco 10270).

### 4.3. Co-immunoprecipitation assay

#### 4.3.1. Transient PEI transfection

HEK 293T cells were seeded at 0.8 × 10^6^ cells per 10 cm^2^ dish and grown for 24 h prior to transfection. A total of 4 µg of the indicated R15 constructs were added to 800 µl of Opti-MEM media (Gibco, 11058) and mixed. Subsequently, 12 µl PEI transfection reagent (Polysciences, 24765) was added, mixed and incubated 20 min at room temperature (RT). The transfection mixture was then added to the 10 cm^2^ cell culture dish drop wise and mixed gently. Cells were incubated for an additional 24 h.

#### 4.3.2. Cell lysates

Cells were gently washed with 5 ml ice-cold PBS before collected and pelleted at 300 RCF for 5 min. The cell pellet was lysed in 800 µl of lysis buffer (50 mM Tris–HCl pH 7.4, 10 mM sodium chloride, 100 mM potassium chloride, 0.1 µM calcium chloride, 0.5 mM magnesium chloride, 0.5 mM TCEP, EDTA-free complete protease tablet (Roche, 04639159001)). The lysates were sonicated on ice for 3 × 3 s using a Microson ultrasonic cell disrupter XL (Misonix), with an output power of 0.06 Watts (RMS Watts). The lysates were clarified by microcentrifugation at 4°C for 12 min at 16 000 RCF. Supernatants were transferred to fresh tubes.

#### 4.3.3. FLAG immunoprecipitation

For each condition, 10 µl anti-FLAG M2 magnetic beads (Sigma-Aldrich, M8823-1 ml), pre-equilibrated in lysis buffer (see above), were added to 700 µl of lysates and incubated overnight at 4°C on a rotating wheel. Samples were washed three times with lysis buffer and proteins were eluted upon addition of 50 µl of 1× BOLT LDS (Novex no. B0007) with 100 mM DTT and boiling at 95°C for 10 min. A total of 10 µl of the immunoprecipitated samples as well as 10 µl of lysates (input) were analysed by immunoblots.

### 4.4. Immunoblotting

Proteins were separated on Bolt 4–12% Bis–Tris Plus gel (Invitrogen, no. NW04120BOX) in 1× MES running buffer. A total of 2 µl of Protein Precision Plus Dual Colour Standards (no. 161-0374) was loaded on each gel. Gels were run at 120 V for 70 min and transferred onto a nitrocellulose membrane (Bio-Rad, 1704159) using a Trans-Blot Turbo System (Bio-Rad). All membranes were stained using Ponceau S (Sigma, P7170) solution for 3 min to assess transfer quality and equal loading. Membranes were then blocked for 1 h at RT using 5% milk in TBS with 0.025% Tween 20 (Sigma, P1379) (TBS-T) with shaking. Membranes were rinsed 3 times with TBS-T and incubated with the relevant primary antibody diluted in 5% BSA in TBS-T overnight at 4°C, while shaking. Membranes were then washed 3 times with TBS-T before incubating with the relevant secondary antibody in TBS-T with 5% milk for 1 h at RT while shaking. Following being washed 3 times with TBS-T and once with TBS, Amersham ECL Prime detection reagent kit (GE Healthcare Life Sciences, RPN2232) was used to detect chemiluminescence with ChemiDoc Touch Imaging System (Bio-Rad).

### 4.5. Activity assay

HEK 293T cells were seeded at 1.5 × 10^6^ to provide overconfluent cells at the time of harvesting. The day after seeding, cells were transfected and lysed as described above.

### 4.6. Antibodies

**Table:** 

protein	antibody	concentration used	species
PP1	Sc-7482	1 : 1000	mouse
total eIF2α	Ab26197	1 : 1500	rabbit
P-eIF2α	Ab32157	1 : 1000	rabbit
vinculin	4650S	1 : 5000	rabbit
FLAG	F7425	1 : 1000	rabbit
actin	20536-1-AP	1 : 10 000	rabbit
anti-rabbit IgG (H + L), HRP conjugate	W4011	1 : 10 000	goat
anti-mouse IgG (H + L), HRP conjugate	W4021	1 : 10 000	goat

## Data accessibility

The data are provided in electronic supplementary material [36].
